# Influence of the center of pressure on baropodometric gait pattern variations in the adult population with flatfoot: A case-control study

**DOI:** 10.3389/fbioe.2023.1147616

**Published:** 2023-03-09

**Authors:** Luis Padrón, Javier Bayod, Ricardo Becerro-de-Bengoa-Vallejo, Marta Losa-Iglesias, Daniel López-López, Israel Casado-Hernández

**Affiliations:** ^1^ Applied Mechanics and Bioengineering Group (AMB), Aragon Institute of Engineering Research (I3A), Universidad de Zaragoza, Zaragoza, Spain; ^2^ Faculty of Nursing, Physiotherapy and Podiatry, Complutense University of Madrid, Madrid, Spain; ^3^ Faculty of Health Sciences, Universidad Rey Juan Carlos, Alcorcón, Spain; ^4^ Research Health and Podiatry Group, Department of Health Sciences, Faculty of Nursing and Podiatry, Industrial Campus of Ferrol, Universidade da Coruña, Ferrol, Spain

**Keywords:** adult flatfoot, gait patterns, musculoskeletal diseases, stance phase gait, foot

## Abstract

**Background:** Adult flatfoot is considered an alteration in the foot bone structure characterized by a decrease or collapse of the medial arch during static or dynamic balance in the gait pattern. The aim of our research was to analyze the center of pressure differences between the population with adult flatfoot and the population with normal feet.

**Methods:** A case-control study involving 62 subjects was carried out on 31 adults with bilateral flatfoot and 31 healthy controls. The gait pattern analysis data were collected employing a complete portable baropodometric platform with piezoresistive sensors.

**Results:** Gait pattern analysis showed statistically significant differences in the cases group, revealing lower levels in the left foot loading response of the stance phase in foot contact time (*p* = 0.016) and contact foot percentage (*p* = 0.019).

**Conclusion:** The adult population with bilateral flatfoot evidenced higher contact time data in the total stance phase compared to the control group, which seems to be linked to the presence of foot deformity in the adult population.

## 1 Introduction

Advances in the quality of life in the adult population have increased life expectancy, and shoe design has evolved during this time (Rao et al., 2015; López-López et al., 2016; Navarro-Flores et al., 2022). Both these factors, which greatly influence foot morphology, have caused an increase in the development of flatfoot in the current population (Saldías et al., 2021). Thus, the flatfoot incidence in the adult population has a developing prevalence of increasing from 26.5% to 29% compared to normal feet (Munro and Steele, 1998; Otsuka et al., 2003).

Adult flatfoot is considered an alteration in the bone foot structure characterized by a decrease or collapse of the medial arch during static or dynamic balance in the gait pattern (Shibuya et al., 2010). Flexible flatfoot is characterized by having a normal arch in non-weight bearing without gait pattern activity or in toe walking and a flattening arch in the static phase. In rigid flatfoot, the arch remains stiff and collapsed with or without weight bearing, and the medial arch is collapsed or shows stiffness in walking (Michaudet et al., 2018). The medial arch is a resistant and elastic link from the synergy of the medial ankle ligaments (deltoid-spring ligament complex), muscle tendons, and plantar fascia (Kitaoka et al., 1997). The spring ligament is the main stabilizer of the medial arch on standing, followed by the deltoid ligament (Brodsky et al., 2009; Orr and Nunley, 2013; Mengiardi et al., 2016; Nery et al., 2018). Furthermore, medial arch stabilization is due to the posterior tibial tendon, which is the main inverter of the midfoot (Mann, 1997).

Posterior tibial tendon dysfunction (PTTD) produces changes in gait patterns resulting in a medial displacement of the center of pressure during the stance phase of gait (Imhauser et al., 2004; Neville et al., 2013; Prachgosin et al., 2015) and a decrease in ankle joint dorsiflexion influenced by decreased soleus muscle activity (Houck et al., 2009; Barn et al., 2013; Lenhart et al., 2014). It can also be argued that PTTD generates changes in the forefoot, increasing abduction and dorsiflexion (Richie, 2007); in the case of the hindfoot, an increase in plantarflexion and eversion is produced in patients with PTTD (Brodsky et al., 2009; Takabayashi et al., 2021).

Nowadays, biomechanical measurement systems of the foot are used to better analyze foot and ankle kinematic gait patterns in every situation (Fritz et al., 2022). For the standing position, the measurement systems commonly used are footprints and radiographs (Lamm et al., 2005; Menz and Munteanu, 2005). Baropodometric platforms measure plantar pressure with the arch index contact force ratio. The plantar pressure measurement and foot structure relationship has been described in previous studies (Teyhen et al., 2009). The arch index has been demonstrated to be an important parameter for studying foot structure and is described as the relation of the midfoot area relative to the total foot area, avoiding the toes (Cavanagh and Rodgers, 1987). Flatfoot measurements are described by an increased arch index, and the arch index contact force ratio is calculated by dividing the contact force on the midfoot area by the total contact force on the total foot area, avoiding the toes (Leung et al., 2004).

The center of pressure (COP) is an important measurement to quantify the force applied to the plantar area of the foot. The COP is commonly known as the gait line during the stance phase and is defined as the spatial distribution of pressure over time represented by a centroid line of each active baropodometric sensor (Cornwall and McPoil, 2000; Landorf and Keenan, 2000). However, the measurement of various features related to stance pattern gait (initial contact phase, forefoot contact phase, and flatfoot phase) and the surface contact foot area (percentage), time foot contact area (milliseconds), and frames foot area (images per second) in people with and without adult flatfoot is unclear.

The aim of our research was to analyze the center of pressure differences between the population with adult flatfoot and the population with normal feet. Our hypothesis was that adults with flatfoot have an increase in the arch index contact, augmenting foot contact regarding normal foot contact without flatfoot subjects and medializing the COP during the stance phase.

## 2 Materials and methods

### 2.1 Design and sample

A total sample of 62 subjects was analyzed in this case-control study (7 men and 55 women). The mean age was 23.48 years old, and the ages of the recruited subjects were between 19 and 34 years old.

The participants of the study were recruited employing a consecutive non-random design in a human movement laboratory of the Universidade da Coruña, in the town of Ferrol (Spain), in the months from May to September 2022 (record number PID2019-108009RB-I00).

Finally, a total of 31 subjects that had developed bilateral flatfoot represented the case group, and the other 31 subjects with healthy common feet were the control group.

For this research the inclusion criteria for the flatfoot group were as follows: 1) to be older than 18 and younger than 64 years old, 2) to be healthy adults without musculoskeletal disorders, foot pain, or significant general health diseases, 3) to be without any lower limb surgery or trauma, 4) to have bilateral flatfoot, 5) to agree to sign the written informed consent form, and 6) to complete all the project stages. The exclusion criteria were as follows: 1) subjects of less than 18 or more than 65 years old, 2) subjects who suffered any relevant foot pain or disturbance, 3) subjects being treated with any medication that could affect the final results, 4) subjects who were pregnant or breastfeeding, 5) subjects who suffered any musculoskeletal disorder or neurological disease, 6) subjects without flatfoot, and 7) subjects that rejected or did not understand the guidelines to take part in the research.

For the control group, the inclusion criteria were as follows: 1) to be older than 18 and younger than 64 years old, 2) to be healthy adults without musculoskeletal disorders, foot pain, or significant general health diseases, 3) to be without any lower limb surgery or trauma, 4) to have bilateral neutral feet, 5) to agree to sign the written informed consent form, and 6) to complete all the project stages. The exclusion criteria were as follows: 1) subjects less than 18 or more than 65 years old, 2) subjects who suffered any relevant foot pain or disturbance, 3) subjects being treated with any medication that could affect the final results, 4) subjects who were pregnant or breastfeeding, 5) subjects who suffered any musculoskeletal disorder or neurological disease, and 6) subjects that rejected or did not understand the guidelines to take part in the research.

### 2.2 Procedure

The study was performed by an expert podiatrist in biomechanical assessment with more than 15 years of experience. At the first visit, subjects were interviewed by the podiatrist, who wrote down the clinical features and global health of the subjects. Then, each subject took off their shoes and socks. Subsequently, the podiatrist checked and recorded anthropometric data, such as height and weight; the body mass index (BMI) was recorded with the subject wearing light clothes and while barefoot and was calculated using Quetelet’s equation for BMI = weight/height^2^ (Macdonald, 1986).

To determine the subjects with flatfoot, the navicular drop (ND) test was performed. Subjects had to stand barefoot on the floor, and the navicular tuberosity was marked by the podiatrist. Next, the talus was placed in a neutral position, palpating the medial and lateral side of the talar dome of the foot with the thumb over the sinus talus and the index over the anteromedial location of the talar dome. The podiatrist performed slowly inverted and everted movements until the talus was settled in a neutral position and the depressions felt under both fingers were the same. Once the subtalar joint was in a neutral position, the distance between the navicular tuberosity and the floor was measured with a ruler and noted in millimeters. Subsequently, the same procedure was repeated in a weight-bearing stance, measuring once again the navicular tuberosity height. The ND was the difference between the two measurement heights. The procedure was repeated three times on each subject (Spörndly-Nees et al., 2011).

In addition to this measurement, a portable baropodometric platform with resistive sensors was used to analyze the normal foot arch (Neo-Plate, Herbitas, Spain), the software being a validated device for foot diagnosis (Painceira-Villar et al., 2021). This study was carried out following the protocol of Becerro de Bengoa Vallejo et al. for recording findings such as dynamic analysis related to the surface area, average COP, body weight on the lower limbs, and foot arch types of each participant in this project (Becerro de Bengoa Vallejo et al., 2013).

### 2.3 Dynamic baropodometric analysis

A complete portable pressure platform with resistive sensors with dual amplifier was used, and automatic multipoint calibration as required for use by the manufacturer was performed before the start of the investigation. The portable platform measured 40 × 40 cm, with a flat surface thickness of 8 mm and a total weight of 4 kg, and comprised 4,096 resistive sensors. Measurements were made to the nearest 0.01 kPa for each sensor. The vertical force was recorded at a frequency of 100–500 Hz. The platform was linked *via* an interface unit to a personal laptop including the data collection computer software Neo-Plate, version for Windows (Herbitas, Foios, Valencia, Spain), and was used according to the protocol stated by Becerro de Bengoa Vallejo et al. for recording findings such as dynamic analysis related to the stance pattern gait (ICP, FFCP, and FFP), surface contact foot area (percentage), time foot contact area (milliseconds), and frames foot area (images per second) (Becerro de Bengoa Vallejo et al., 2013).

The COP locus area (% CLA) is defined by the area ratio embraced by the COP path and a line between the start and the end points of the COP path to the foot area. For the research, the frames and percentages in each stance pattern gait were acquired. The initial contact phase (ICP) corresponded to the loading response of the stance phase and began with initial floor contact and continued until the other foot was lifted for the swing. The forefoot contact phase (FFCP) corresponded to the total stance of the stance phase, and the foot was in total contact with the ground; and finally, the flatfoot phase (FFP) corresponded to the final phase of the stance when the toe-off occurred. The dynamic was created for each foot variable by including 1) surface contact foot area (percentage), 2) time foot contact area (milliseconds), and 3) frames foot area (images per second).

### 2.4 Sample size calculation

To determine the sample size, G* Power 3.1.9.3 software (Heinrich-Heine-Universität Düsseldorf, Germany) was used to test the correlation between two paired means regarding correspondence with a Spearman correlation coefficient of 0.40 and a 95% confidence interval (CI) for a two-tailed test, an α error of 0.05, and an estimated analysis power of 80% (error *β* = 20%). For all the analyses, the minimum sample size was 62 participants (31 per group).

### 2.5 Ethical and legal considerations

This study was carried out from May to September 2022 and followed all the criteria of the Strengthening the Reporting of Observational Studies in Epidemiology (STROBE) guidelines (Vandenbroucke et al., 2014). The study was accepted by an ethics committee, and all the actions were taken according to the ethical standards for human research presented in the Declaration of Helsinki (Shrestha and Dunn, 2020). In addition, subjects were recruited by a human movement laboratory at the Universidade da Coruña, in the town of Ferrol (Spain), and took part in the project with record number PID2019-108009RB-I00, which received approval from the Research Ethics Committee at the University of A Coruña, Spain; file number 2019-0017; date: 6 November 2019.

### 2.6 Statistical analysis

The IBM SPSS Statistics 27.0.01.0 package for windows (Armonk, NY, United States) was applied for the analysis of the outcomes in this research. In all the analyses, significance was established at *p* < 0.05 with a 95% confidence interval.

Normality was checked using the Kolmogorov–Smirnov test of the variables studied (*p* > 0.05) on the data on static plantar measurements. The results of the independent Student’s t-tests were used to decide if the data were normally distributed, and parametric statistical tests were found to be the most appropriate. The non-parametric Mann–Whitney “U” test was performed to consider contrasts among the two groups with or without adult flatfoot.

The independent variables are shown as mean, ranges of minimum to maximum, and standard deviation values for the descriptive data analysis. Concerning the categorical variables, they are presented as percentages and absolute values. The software Neo-Plate, version for Windows, was used to obtain the stance pattern gait (ICP, FFCP, and FFP) and the surface contact foot area (percentage), time foot contact area (milliseconds), and frames foot area (images per second) that were generated for each foot with or without adult flatfoot.

## 3 Results

### 3.1 Sociodemographic data

A total sample of 62 subjects, between 19 and 34 years of age, with a mean age ± SD of 23.48 ± 5.46 years, completed all the research. Most voluntary participants were overweight, BMI of 26.51 ± 5.61 kg/m^2^, with statistically significant differences (*p* < 0.001). The main descriptive characteristics of all the subjects, as well as stratified by groups with or without bilateral adult flatfoot, are described in Table 1.

**TABLE 1:** Main characteristics of the total sample with or without bilateral adult flatfoot.

Characteristics	Total sample (*n* = 62)	Case group (*n* = 31)	Control group (*n* = 31)	*p*-value
	Mean ± SD (range)	Mean ± SD (range)	Mean ± SD (range)	
Age (years)	23.48 ± 5.46 (19–34)	23.87 ± 4.23 (19–34)	23.10 ± 4.21 (19–34)	0.097^a^
Weight (kg)	71.26 ± 14.58 (48–98)	77.36 ± 15.10 (56–98)	65.16 ± 11.28 (48–89)	**< 0.001** ^a^
Height (cm)	164.69 ± 7.44 (152–185)	163.13 ± 6.54 (152–175)	166.26 ± 8.05 (155–185)	0.138^a^
BMI (kg/m^2^)	26.51 ± 5.61 (19.00–39.26)	29.25 ± 6.29 (21.08–39.26)	23.76 ± 2.97 (19.00–30.48)	**< 0.001** ^a^
Sex, male/female (%)	7/55 (11.3/88.7)	4/27 (12.9/87.41)	3/28 (9.7/90.3)	1.000^b^
Foot size	38.84 ± 2.15 (36–46)	38.55 ± 1.71 (36–42)	39.12 ± 2.51 (36–46)	0.436^a^

Kg, kilogram; Cm, centimeter; % percentage; SD, standard deviation; N, number.

^a^ Mann–Whitney *U* test was used.

^b^ Fisher’s exact test was used.

In all the analyses, *p* < .05 (with a 95% confidence interval) was considered statistically significant (in bold).

### 3.2 Main outcome measures data

The main findings are described in Table 2. When the gait analysis of adult flatfoot was performed, we observed that left foot minimum frame FFP was lower in the case group than in the control group. There was also a difference between groups in time, percentage, and maximum frame in left foot ICP.

**TABLE 2:** Main outcome measurements of gait analysis of the total sample with or without bilateral adult flatfoot.

Characteristics	Total sample (*n* = 62)	Case group (*n* = 31)	Control group (*n* = 31)	*p*-value
	Mean ± SD (range)	Mean ± SD (range)	Mean ± SD (range)	
Left foot FFCP (ms)	240.27 ± 55.82 (157–414)	241.29 ± 43.39 (178–306)	239.26 ± 55.82 (157–414)	0.582^a^
Left foot FFCP (%)	33.06 ± 7.13 (20–48)	33.03 ± 5.97 (21–43)	33.10 ± 8.22 (20–48)	0.955^a^
Left foot min. frame FFCP	51.95 ± 8.83 (34–73)	52.61 ± 8.93 (41–73)	51.29 ± 8.81 (34–70)	0.921^a^
Left foot max. frame FFCP	76.32 ± 7.53 (58–91)	77.06 ± 7.41 (68–91)	75.58 ± 7.70 (58–89)	0.601^a^
Left foot FFP (ms)	426.74 ± 91.57 (275–632)	445.32 ± 101.56 (305–632)	408.16 ± 77.60 (275–593)	0.064^a^
Left foot FFP (%)	56.10 ± 8.88 (38–70)	57.68 ± 9.26 (43–70)	54.52 ± 8.32 (38–69)	0.191^a^
Left foot min. frame FFP	8.61 ± 3.75 (1–13)	7.42 ± 4.09 (1–13)	9.81 ± 2.98 (3–13)	**0.012** ^a^
Left foot max. frame FFP	51.95 ± 8.83 (34–73)	52.61 ± 8.93 (41–73)	51.29 ± 8.82 (34–70)	0.921^a^
Left foot ICP (ms)	84.26 ± 37.03 (9–128)	72.55 ± 40.48 (9–128)	95.97 ± 29.45 (29–128)	**0.016** ^a^
Left foot ICP (%)	10.84 ± 4.91 (1–18)	9.29 ± 5.64 (1–18)	12.39 ± 3.50 (4–18)	**0.019** ^a^
Left foot min. frame ICP	0 ± 0.00 (0–0)	0 ± 0.00 (0–0)	0 ± 0.00 (0–0)	1.000^a^
Left foot max. frame ICP	8.71 ± 3.89 (1–15)	7.42 ± 4.09 (1–13)	10.00 ± 3.25 (3–15)	**0.009 †**
Right foot FFCP (ms)	273.02 ± 151.21 (148–748)	293.74 ± 192.99 (148–748)	252.29 ± 91.40 (158–455)	0.871^a^
Right foot FFCP (%)	37.37 ± 19.23 (20–99)	39.65 ± 24.79 (21–99)	35.10 ± 11.26 (20–58)	0.849^a^
Right foot min. frame FFCP	48.66 ± 16.30 (1–71)	47.29 ± 20.18 (1–71)	50.03 ± 11.36 (30–68)	0.799^a^
Right foot max. frame FFCP	76.39 ± 8.62 (54–91)	77.10 ± 7.50 (68–89)	75.68 ± 9.68 (54–91)	0.827^a^
Right foot FFP (ms)	398.15 ± 139.89 (0–602)	379.06 ± 171.40 (0–602)	417.23 ± 98.28 (254–561)	0.719^a^
Right foot FFP (%)	52.47 ± 16.88 (0–70)	49.35 ± 21.02 (0–68)	55.58 ± 10.84 (34–70)	0.520^a^
Right foot min. frame FFP	8.18 ± 3.56 (1–14)	8.74 ± 3.29 (1–11)	7.61 ± 3.78 (1–14)	0.099^a^
Right foot max. frame FFP	48.66 ± 16.30 (1–71)	47.29 ± 20.18 (1–71)	50.03 ± 11.36 (30–68)	0.799^a^
Right foot ICP (ms)	80.00 ± 35.23 (9.00–138)	85.55 ± 32.55 (9.00–108)	74.45 ± 37.43 (9.00–138)	0.099^a^
Right foot ICP (%)	10.16 ± 4.66 (1–16)	11.00 ± 4.58 (1–16)	9.32 ± 4.66 (1–15)	0.171^a^
Right foot min. frame ICP	0 ± 0.00 (0–0)	0 ± 0.00 (0–0)	0 ± 0.00 (0–0)	1.000^a^
Right foot max. frame ICP	8.18 ± 3.56 (1–14)	8.74 ± 3.29 (1–11)	7.61 ± 3.78 (1–14)	0.099^a^

ICP, initial contact phase; FFCP, forefoot contact phase; FFP, flatfoot phase; Ms, meters per second; Kpa, kilopascal; Kg, kilogram; Cm, centimeter; % percentage; SD, standard deviation; N, number.

^a^ Mann–Whitney *U* test was used.

In all the analyses, *p* < .05 (with a 95% confidence interval) was considered statistically significant (in bold).

There were no statistically significant differences between groups in the gait analysis supported by the lower left and right limbs.

## 4 Discussion

This research is the first to show alterations in gait analysis in the adult population with bilateral flatfoot compared to healthy individuals. These changes in stance pattern gait can be attributed to bilateral foot conditions, which leads to flattening of the medial arch in the foot in weight-bearing and can be the cause of lack of a propulsive walk and alterations in time, percentage, and minimum or maximum frames, in all the phases of the gait.

Thus, the aim of our research was to analyze the center of pressure differences between the population with adult flatfoot and the population with normal feet. This procedure was carried out according to the protocol of previous studies (Becerro de Bengoa Vallejo et al., 2013) for recording time, percentages, and frames from the subjects in this project.

However, the results of our findings showed statistically significant differences in the initial contact phase (ICP) in the left foot between the two groups. The case group decreased the contact area in time and percentage. Anyway, characteristics of adult flatfoot are directly related to collapse of the longitudinal arch, hindfoot valgus, and forefoot abduction (Filardi, 2018). The load bearing distinctive of the ankle and foot complex in the stance phases demands determined muscular loading to bear the longitudinal arch. Tissue suffering may happen in determined foot areas, concretely on the plantar foot, and should exhibit different stiffness degrees (Filardi, 2018). According to our findings, an increase in the foot time and an increase in the contact percentage in both feet in the total contact stance phase were observed in the case group versus the control group.

It is not easy to compare the influence of these outcomes with previous studies due to the discrepancies in exclusion and inclusion criteria of the procedures and methodological differences, as we have not been capable of finding research relating to stance pattern gait (ICP, FFCP, and FFP) and the surface contact foot area (percentage), time foot contact area (milliseconds), and frames foot area (images per second) in adults with or without bilateral flatfoot.

However, based on the findings of the previous investigations carried out on this topic, we found that Fan et al. compared natural gait in subjects with flatfoot and subjects with an increased medial arch and showed that vertical ground reaction force of the plantar brings greater muscle tension to the flat-footed and a smaller rate of change of footprint area recording greater stability to the high-arched. The results of their findings showed an increase in the percentage of stance phase in subjects with flatfoot (61.034%) versus subjects with a high medial arch (60.784%), but they did not differentiate between right and left feet (Fan et al., 2011). In our findings, we found an increase in the case group right foot FFCP (%) (39.65%) versus the control group right foot FFCP (%) (35.10%).

Jankowicz-Szymańska et al. analyzed the foot longitudinal arch height in overweight adults and concluded that a high weight was correlated with a decreased height of the medial arch and an excessive body weight contributed to the progression of flatfoot despite age (Jankowicz-Szymańska et al., 2018). According to our research, the case group’s BMI was 29.25 kg/m^2^, regardless of age, and all the participants presented flatfoot.

We observe some limitations in our research. The baropodometric platform measurement portable system can only record and identify vertical force at a frequency of 60 Hz. Other frequencies and different forces could be relevant in the capturing and recording of force movement on the foot sole, such as shearing stress and pressure on the feet; these were not represented. Moreover, related biomechanical musculoskeletal lower limb gait pattern data, such as electromyography and kinematics parameters, were not recorded, so it is difficult to establish conclusions about these effects on the flatfoot gait parameters in every stance phase of the gait. However, this novel case-control research provides advantageous knowledge on usual foot diseases to clinicians and researchers about stance phase gait parameters in the adult population with flatfoot deformities. Furthermore, it reveals the significance of continuous investigation related to adult flatfoot and its assessment to improve the diagnosis and outcome of foot health problems and people’s quality of life.

## 5 Conclusion

The findings of this research show alterations in gait analysis in the adult population with bilateral flatfoot compared to healthy individuals. Specifically, the patients with bilateral foot problems evidenced that left foot minimum frame FFP was lower in the case group than in the control group. There was also a difference between groups in time, percentage, and maximum frame in left foot ICP, which seems to be linked with the presence of foot deformity in the adult population.

## Data Availability

The raw data supporting the conclusion of this article will be made available by the authors, without undue reservation.
